# Patient sex and use of tranexamic acid in liver transplantation

**DOI:** 10.3389/fmed.2024.1452733

**Published:** 2024-09-23

**Authors:** Sarah Dehne, Lorena Jackson-Gil, Carlo Riede, Manuel Feisst, Arianeb Mehrabi, Christoph W. Michalski, Markus A. Weigand, Sebastian O. Decker, Jan Larmann

**Affiliations:** ^1^Heidelberg University, Medical Faculty Heidelberg, Department of Anesthesiology, Heidelberg, Germany; ^2^Heidelberg University, Institute of Medical Biometry, Heidelberg, Germany; ^3^Heidelberg University, Medical Faculty Heidelberg, Department of General, Visceral, and Transplantation Surgery, Heidelberg, Germany

**Keywords:** tranexamic acid, sex-specific differences, liver transplantation, perioperative antifibrinolytic management, postoperative complications

## Abstract

**Background:**

Differences in medical treatment between women and men are common and involve out-of-hospital emergency care, the intensity of pain treatment, and the use of antifibrinolytic treatment in emergency trauma patients. If woman and man receive different antifibrinolytic treatment in highly-standardized major transplant surgery is unknown.

**Methods:**

We conducted a retrospective cohort study on patients who underwent liver transplantation at Heidelberg University Hospital, Heidelberg, Germany between 2004 and 2017. Logistic regression analyses were performed to determine if sex is associated with the administration of TXA during liver transplantation. Secondary endpoints included venous thrombotic complications, graft failure, mortality, myocardial infarction, hepatic artery thrombosis, and stroke within the first 30 days after liver transplant as well as length of hospital stay and length of intensive care unit stay.

**Results:**

Out of 779 patients who underwent liver transplantation, 262 patients received TXA. Female sex was not associated with intraoperative administration of TXA [adjusted OR: 0.929 (95% CI 0.654; 1.320), *p* = 0.681]. The secondary endpoints graft failure (13.2% vs. 8.4%, women vs. men, *p* = 0.039), pulmonary embolism (3.4% vs. 0.9%, women vs. men, *p* = 0.012), stroke (1.7% vs. 0.4%, women vs. men, *p* = 0.049), and deep vein thrombosis (0.8% vs. 0%, women vs. men, *p* = 0.031) within 30 days after liver transplantation were more frequent in women. Mortality, myocardial infarction, and other secondary endpoints did not differ between groups. However, in women, the use of TXA was associated with a lower rate in thromboembolic complications.

**Conclusion:**

Our data indicate that different from other scenarios with massive bleeding complications the administration of TXA during liver transplantation is not associated with sex. However, sex is associated with the risk for complications, and in woman TXA might have a preventive effect on the rate of thromboembolic complications. Reasons underlying the observed sex bias rate remain uncertain.

## 1 Introduction

Differences in medical treatment between women and men are common and involve out-of-hospital emergency care, imaging diagnostics in major trauma, the intensity of pain treatment, and the use of antifibrinolytic treatment in emergency trauma patients (1–4). Women with bleeding trauma are treated less frequently with tranexamic acid (TXA) than men, although the administration of TXA reduces mortality to a comparable degree in both (4). Nutbeam et al. analyzed data from the Trauma and Audit Research Network for 216,364 patients aged over 16 years with an Injury Severity Score greater than 9, comprising 98,879 (46%) females and 117,485 (54%) males. Among them, 7,198 (7.3%) of females and 19,697 (16.8%) of males received TXA (OR = 0.39 [0.38–0.40]) (4). A sex disaggregated analysis was also performed in 32,948 patients of data from the Clinical Randomization of an Antifibrinolytic in Significant Hemorrhage (CRASH)-2 and CRASH-3 trials. TXA lowered the risk of death equally in women and men [women: OR = 0.69 (0.52–0.91), men: OR = 0.80 (0.71–0.90); *p* = 0.34] (4).

TXA is commonly used during liver transplantation with the aim of reducing blood loss or the need for blood transfusions (5–7). TXA, a synthetic lysine analogue, exerts antifibrinolytic effects by binding to the lysine-binding sites of plasminogen. This binding prevents the interplay between plasmin and fibrin (8). The decision to administer TXA is subject to a comprehensive evaluation including clinical bleeding assessment, rotational thrombelastometry and laboratory results and is ultimately at the discretion of the supervising anesthesiologist (9).

The potential sex-specific differences in the use of tranexamic acid during liver transplantation have been poorly investigated. In a small retrospective study examining 162 patient records on the characteristics and outcomes of liver transplant recipients following tranexamic acid treatment and platelet transfusion, no differences in the distribution of sexes were observed between the TXA and non-TXA groups (10). However, whether the decision to administer TXA during liver transplantation is made regardless of sex, is unclear. Therefore, we conducted the current study to determine whether sex is associated with the administration of TXA during liver transplantation.

## 2 Methods

### 2.1 Study design

A retrospective cohort study was undertaken on adult liver transplant recipients at Heidelberg University Hospital, Heidelberg, Germany from 2004 to 2017, to determine if there is an association between sex and the administration of TXA during liver transplantation. Adult patients undergoing liver transplantation were considered for inclusion in this study if their anesthesia records were accessible and contained comprehensive data on outcomes and baseline parameters. Patients in need for retransplantations were excluded from the analysis if the last known previous transplant occurred less than 30 days before the retransplantation under investigation or if the date was unavailable. Additionally, patients who underwent retransplantation during the same hospital stay or who died during or within 1 day after surgery were excluded. Our study protocol (S-070/2024) received approval by the local Ethics Committee of the Medical Faculty of Ruprecht-Karls-University, Heidelberg, Germany, on 01 March, 2024. The principles described in the Declaration of Helsinki and the STROBE guidelines for observational studies have been followed in conducting this study (11).

### 2.2 Procedures

General anesthesia and surgical procedures were conducted in accordance with departmental standards. For anesthesia induction propofol and sufentanil or remifentanil was used, sevoflurane or the less commonly used desflurane combined with remifentanil were used for maintenance. Liver transplantation follows the modified piggyback technique as per Belghiti’s method, which involves a cavo-caval side-to-side anastomosis (12).

### 2.3 Data collection

Recipient and allocated donor data were extracted from the electronic patient records. These included: demographic data, weight, laboratory parameters, pre-existing conditions, Child-Pugh-Score, Model of End-stage Liver Disease (MELD)-score, prior abdominal surgery, retransplantation, cold ischemia time, and duration of surgery. Intraoperative blood loss data and transfusion requirement were gathered from anesthesia records.

### 2.4 Outcome analysis

The pre-specified primary outcome was administration of TXA during liver transplantation. TXA administration was quantified in grams and retrieved from anesthesia records. The decision to administer tranexamic acid involved a comprehensive evaluation including clinical bleeding assessment based on the surgeon’s experience, evaluation of rational thrombelastometry (ROTEM^®^) results as outlined in the internal standard operating procedures (Supplementary Table S1), and was ultimately at the discretion of the attending anesthesiologist. Pre-specified secondary endpoints were, graft failure defined as the failure of the liver allograft necessitating retransplantation or resulting in the recipient’s death, deep vein thrombosis, 30-day mortality, myocardial infarction, pulmonary embolism, hepatic artery thrombosis, portal vein thrombosis and stroke within the initial 30 days post-liver transplant, alongside the duration of hospitalization and intensive care unit (ICU) stay. Furthermore, a subgroup analyses was performed for the secondary endpoints, considering the most common underlying diseases that led to the liver transplantation in this cohort.

Additionally, we investigated whether thromboembolic events were more common in women and men who received intraoperative TXA compared to those who did not. The thromboembolic events were examined in a composite endpoint comprising pulmonary embolism, myocardial infarction, stroke and deep vein thrombosis, as well as the individual components of the composite endpoint.

### 2.5 Statistical analysis

The patient cohort was split into two groups according to sex. Thereafter, the patient cohorts were further divided depending on TXA administration. Various factors, such as aggravated surgical conditions or the severity of liver disease, which are likely to lead to bleeding tendencies where hyperfibrinolysis is anticipated and May favor intraoperative administration of tranexamic acid (7, 13–15), and baseline characteristics that differed between groups (Tables 1, 2) were analyzed as covariates using univariable analysis in a logistic regression model: Female sex, age, and BMI of both donor and recipient, recipient/donor weight ratio, MELD-score, transplant priority, previous abdominal surgeries, duration of surgery, cold ischemia time, retransplantation, Child-Pugh-score, preoperative hemoglobin, preoperative platelet count, and blood loss. To achieve a more inclusive model, all covariates with *p*-values below 0.1 in the univariable analysis were chosen as covariates for the multivariable analysis. Since sex is the variable of interest, it was also included in the multivariable regression model regardless of the univariate analysis results. Thus, a more precise evaluation of sex is achieved by considering other influencing factors and potential interactions, and by revealing any effects of sex that might be obscured by other variables. A significance level of *p* < 0.05 was considered. To compare continuous data, the Mann–Whitney U-test or the *t*-test was used as appropriate. Comparison of categorical data was conducted using the chi-square test. IBM SPSS Statistics 28.0 (SPSS, Chicago, IL) was used to perform statistical analyses.

**Table 1:** Baseline characteristics of the study cohort.

Variable	Analysis set 779 (100)	Female sex 234 (30)	Male sex 545 (70.0)	*p* value
***Recipient***				
Age (y), mean ± SD	52.2 ± 10.3	49.6 ± 11.3	53.3 ± 9.7	**<0.001**
Weight (kg), mean ± SD	80.2 ± 16.6	70.9 ± 15.4	84.1 ± 15.4	**<0.001**
BMI (kg m^−2^), mean ± SD	26.6 ± 4.8	25.9 ± 5.2	26.9 ± 4.6	**0.004**
Retransplantation, *n* (%)	67 (8.6)	16 (6.8%)	51 (9.4%)	0.250
*Child-Pugh, n (%)*				
A	221 (28.4)	68 (29.0)	153 (28.1)	**<0.001**
B	214 (27.5)	41 (17.5)	173 (31.7)	
C	344 (44.2)	125 (53.4)	219 (40.1)	
MELD, mean ± SD	19.3 ± 10.4	20.5 ± 11.1	18.7 ± 10.0	0.095
***Urgency, n (%)***				
T	736 (94.5)	204 (87.1)	532 (97.6)	**<0.001**
HU	43 (5.5)	30 (12.8)	13 (2.3)	
***Medical history, n (%)***				
Previous deep vein thrombosis	11 (1.4)	4 (1.7)	7 (1.2)	0.645
Previous hepatic artery thrombosis	11 (1.4)	5 (2.1)	6 (1.1)	0.261
Previous portal vein thrombosis	84 (10.8)	27 (11.5)	57 (10.5)	0.656
Other thrombosis in history	107 (13.7)	37 (15.8)	70 (12.8)	0.270
Previous abdominal surgery, *n* (%)	275 (35.3)	104 (44.4)	171 (31.3)	**<0.001**
Coronary heart disease, *n* (%)	66 (8.47)	13 (5.5)	53 (9.7)	0.055
History of myocardial infarction, *n* (%)	20 (2.567)	4 (1.7)	16 (2.9)	0.321
History of stroke, *n* (%)	6 (0.77)	1 (0.4)	5 (0.9)	0.473
***Donor***				
Age (y), mean ± SD	58.1 ± 17.3	57 ± 19	59 ± 16	0.453
Female sex, *n* (%)	359 (46.1)	148 (63.2)	211 (38.7)	**<0.001**
Weight (kg), mean ± SD	77.5 ± 15.0	69.9 ± 15.2	80.7 ± 13.8	**<0.001**
BMI (kg m^−2^), mean ± SD	26.3 ± 6.1	25.3 ± 8.9	26.8 ± 4.3	**<0.001**
Weight ratio recipient/donor, mean ± SD	1.1 ± 0.3	1.1 ± 0.3	1.1 ± 0.2	0.030
***Surgery data***				
Preoperative hemoglobin (g/dl), mean ± SD	10.9 ± 2.6	10.5 ± 2.8	11.0 ± 2.5	**0.001**
Preoperative platelet count, mean ± SD	119.5 ± 91.3	128.9 ± 97.7	115.4 ± 88.2	0.178
Intraoperative blood loss (ml/kg), mean ± SD	55.27 ± 57.6	55.96 ± 55.6	54.5 ± 58.5	0.964
Cold ischemia time (h), mean ± SD	8.6 ± 3.1	8.23 ± 3.4	8.75 ± 2.9	0.117
Surgery time (h), mean ± SD	5.8 ± 1.5	5.7 ± 1.53	5.9 ± 1.4	0.**038**

Data are presented as mean ± SD, or as absolute number (percentage). SD, standard deviation; BMI, body mass index; MELD, Model for End-stage Liver Disease; T, transplantable; HU, high urgency. Bold face indicates *p*-values < 0.05.

**Table 2:** Logistic regression on primary endpoint.

Variable	Univariable		Multivariable	
	OR (95% CI)	*p*-value	Adjusted OR (95% CI)	*p*-value
Female recipient	0.878 (0.633; 1.218)	0.437	0.929 (0.654; 1.320)	0.681
Age recipient	1.008 (0.993; 1.023)	0.284		
BMI recipient	1.006 (0.975; 1.037)	0.713		
Female donor	0.816 (0.605; 1.101)	0.184		
Age donor	1.003 (0.994; 1.011)	0.529		
BMI donor	1.033 (1.001; 1.066)	0.045	1.030 (0.998; 1.063)	0.065
Weight ratio recipient/donor	0.919 (0.490; 1.723)	0.791		
History of abdominal surgery	1.120 (0.822; 1.526)	0.474		
Cold ischemia time	1.040 (0.990; 1.093)	0.120		
MELD-Score	1.029 (1.015; 1.044)	<0.001	1.031 (1.008; 1.055)	**0.008**
Retransplantation	0.999 (0.997; 1.002)	0.574		
Surgery time	1.159 (1.047; 1.283)	0.004	1.116 (0.997; 1.250)	0.057
Preoperative hemoglobine	0.913 (0.860; 0.970)	0.003	0.957 (0.888; 1.030)	0.242
Preoperative platelet count	0.997 (0.995; 0.999)	0.003	0.998 (0.996; 1.000)	0.059
Blood loss	1.000 (1.000; 1.000)	0.005	1.000 (1.000; 1.000)	0.408
*Child-Pugh*				
A (reference)		0.118		0.107
B	0.701 (0.485; 1.011)	0.057	1.679 (0.970; 2.905)	0.064
C	1.010 (0.708; 1.441)	0.957	1.545 (0.993; 2.402)	0.054
*Urgency*				
HU	0.664 (0.329; 1.340)	0.253		

OR estimated from the logistic regression analysis were reported with corresponding 95% CIs. Bold face indicates *p*-values < 0.05 in multivariable proportional hazards analysis. OR, odds ratio; CI, confidence interval; BMI, body mass index; MELD, Model for End-stage Liver Disease; T, transplantable; HU, high urgency.

## 3 Results

**Figure 1:**
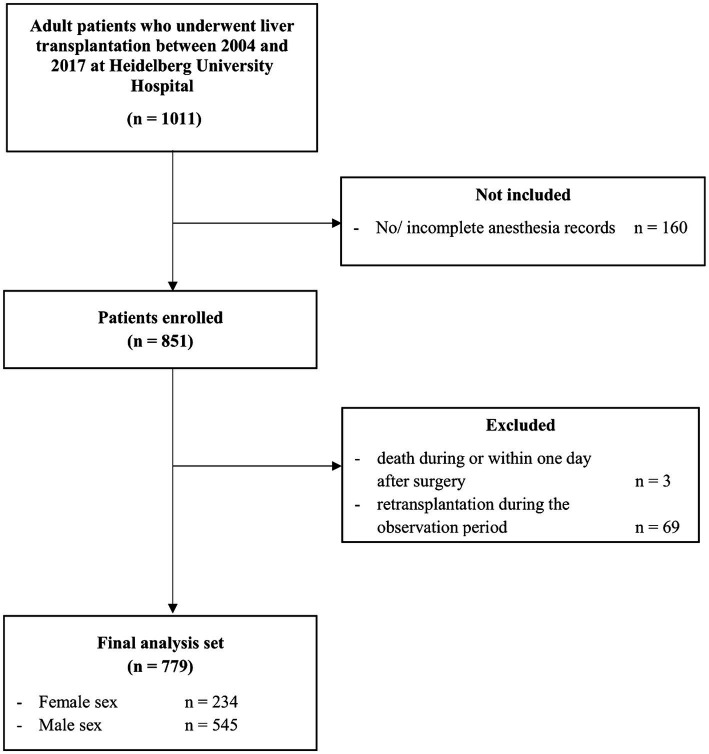
Participant flow chart.

Data from 1,011 patients were included during the observation period. After applying the inclusion and exclusion criteria, 779 patients who underwent liver transplantation were selected for final analyses (Figure 1).

### 3.1 Patient characteristics

Baseline characteristics of the patient cohort are displayed in Table 1. The average age of the recipients was 52 ± 10 years, and their average BMI was 26.6 ± 4.8 kg m^−2^. The majority of patients had advanced-stage Child-Pugh C liver cirrhosis. The average MELD score for the recipients was 19.3 ± 10.4. At the time of transplantation, 275 patients (35.3%) had a history of previous abdominal surgery. A total of 67 patients (8.6%) underwent retransplantation. The average age of the donor was 58 ± 17 years, and their average BMI was 26.3 ± 6.1 kg m^−2^. 46.1% of the donors were female. The average cold ischemia time for the donor organs was 9 ± 3 h. The average transplantation duration was 6 ± 2 h. The female group contained 234 patients and the male group compromised 545 patients. The underlying liver diseases that led to liver transplantation differed between women and men. In women, biliary tract disease (19.7%) was the most prevalent underlying liver disease, followed by alcoholic liver cirrhosis (18.8%), hepatitis (16.2%) and acute liver failure (14.1%). In men, the most common liver disease was alcoholic liver cirrhosis (30.6%), followed by hepatitis (23.9%), biliary tract diseases (15.2%) and malignant liver tumors (11.6%) (Supplementary Table S2A). Age, weight and BMI were lower in the female group. In the group of women, the allocated donors were more frequently female. Additionally, the weight and BMI of the donors were lower in the female group. At the time of transplantation, women were more likely to have undergone previous abdominal surgery, exhibited a higher Child-Pugh score, and were more frequently listed with High Urgency priority compared to men. They also had lower hemoglobin levels and underwent slightly shorter surgery times compared to men. Fresh frozen plasma was transfused more frequently in men (90.8%) with a dosage of 18 ± 13 transfusion units (TU) than in women (86.8%) with a dosage of 16 ± 11 TU. The transfusion rate of other blood products or coagulation factor administration did not differ between the groups (Supplementary Table S3). The patient cohort was additionally compared in relation to intraoperative administration of TXA (Supplementary Table S4). TXA administration was more frequent in patients with retransplantation. Patients who received intraoperative TXA had a higher MELD score, and were more likely to have a history of thrombosis, excluding deep vein thrombosis, portal vein thrombosis, or hepatic artery thrombosis. They also had lower hemoglobin levels and platelet counts at the time of transplantation, higher intraoperative blood loss, a longer cold ischemia time and a longer surgery time.

### 3.2 Sex is not associated with the intraoperative administration of tranexamic acid

Out of the entire cohort, intraoperative TXA was administered to 262 patients (33.6%). The average dose was 1.4 ± 0.7 g. Women did not receive intraoperative TXA more or less frequently compared to men (31.6% vs. 34.5%, female vs. male, *p* = 0.437). Women received an average dose of 1.5 ± 0.7 g compared to men with an average dose of 1.4 ± 0.7 g (*p* = 0.139). Since there are considerable weight differences between women and men, the dose was again compared as a function of body weight. On average, women received a higher dose of TXA in relation to their weight than men (21.6 ± 10.8 mg kg^−1^ vs. 17.1 ± 9.8 mg kg^−1^, women vs. men, *p* < 0.001). In univariable analysis, female sex was not correlated with intraoperative TXA administration [OR 0.878 (95% CI 0.633; 1.218), *p* = 0.437] (Table 2).

In the univariable analysis, seven variables with *p*-values <0.1 were identified and integrated into the multivariable logistic regression model alongside female sex: BMI of the donor, MELD-score, surgery time, preoperative hemoglobin, preoperative platelet count, blood loss and Child-Pugh-Score (Table 2).

In the multivariable analysis, female sex consistently showed no association with the administration of TXA [adjusted OR: 0.929 (0.654; 1.320), *p* = 0.681]. The MELD-score was the only independent predictor for TXA administration during liver transplantation [OR 1.031 (95% CI 1.008; 1.055), *p* = 0.008] (Table 2).

### 3.3 Secondary endpoints

In total, women were more likely to suffer graft failure (13.2% vs. 8.4%, women vs. men, *p* = 0.039) pulmonary embolism (3.4% vs. 0.9%, women vs. men, *p* = 0.012), stroke (1.7% vs. 0.4%, women vs. men, *p* = 0.049) and deep vein thrombosis (0.8% vs. 0%, women vs. men, *p* = 0.031) than men within 30 days after liver transplantation. There were no differences observed between women and men in terms of the occurrence of hepatic artery thrombosis, portal vein thrombosis, myocardial infarction, or 30-day mortality. Also, length of ICU stay and length of hospital stay did not differ between men and women (Table 3). A subgroup analysis was performed for patients with hepatitis, alcoholic liver cirrhosis, malignant liver diseases, and cholangitis. In women with alcoholic liver cirrhosis, hepatic artery thrombosis occurred more frequently than in men [4 (9.1%) vs. 4 (2.4%), women vs. men, *p* = 0.039]. In women with hepatitis, pulmonary embolism was more common than in men [2 (5.3) vs. 0 (0), women vs. men, *p* = 0.009]. For the other secondary endpoints, no differences were observed between men and women within the respective subgroups (Supplementary Table S2B). In women, intraoperative administration of TXA was associated with a lower incidence of the composite endpoint for thromboembolic complications. However, the individual components of this composite endpoint were not associated with female sex. The incidence of thromboembolic complications in men was not affected by intraoperative administration of TXA (Table 4).

**Table 3:** Postoperative complications and data of hospital stay.

Variable	Analysis set (*n* = 779)	Female sex (*n* = 234)	Male sex (*n* = 545)	*p* value
30-day-mortality, *n* (%)	45 (5.8)	18 (7.7)	27 (5.0)	0.133
Graft failure, *n* (%)	77 (9.9)	31 (13.2)	46 (8.4)	**0.039**
Pulmonary embolism, *n* (%)	13 (1.7)	8 (3.4)	5 (0.9)	**0.012**
Myocardial infarction, *n* (%)	14 (1.8)	3 (1.2)	11 (2.0)	0.478
Stroke, *n* (%)	6 (0.8)	4 (1.7)	2 (0.4)	**0.049**
Deep vein thrombosis, *n* (%)	2 (0.3)	2 (0.8)	0 (0)	**0.031**
Hepatic artery thrombosis, *n* (%)	46 (5.9)	20 (8.5)	26 (4.8)	0.103
Portal vein thrombosis, *n* (%)	24 (3.1)	10 (4.3)	14 (2.6)	0.127
Length of hospital stay (d), median (Q1, Q3)	32 (22;51)	33 (23;53)	31 (21;50)	0.111
Length of ICU stay (d), median (Q1, Q3)	3 (1;9)	4 (2;10)	3 (1;8)	0.079

Data are presented as absolute numbers (percentage) or mean ± SD. Bold face indicates *p*-values < 0.05. ICU, intensive care unit; SD, standard deviation.

**Table 4:** Postoperative thromboembolic complications in women and men.

Variable	Analysis set	Intraoperative TXA	No intraoperative TXA	*p* value
*Women, n*	*234*	*74*	*160*	
Thromboembolic complications, *n* (%)	17 (7.2)	1 (1.3)	16 (10)	**0.018**
Pulmonary embolism, *n* (%)	8 (3.4)	0 (0)	8 (5.0)	0.050
Myocardial infarction, *n* (%)	3 (1.2)	0 (0)	3 (1.9)	0.236
Stroke, *n* (%)	4 (1.7)	1 (1.4)	3 (1.9)	0.774
Deep vein thrombosis, *n* (%)	2 (0.8)	0 (0)	2 (1.3)	0.334
*Men, n*	*545*	*188*	*357*	
Thromboembolic complications, *n* (%)	18 (3.3)	4 (2.1)	14 (3.9)	0.265
Pulmonary embolism, *n* (%)	5 (0.9)	1 (0.5)	4 (1.1)	0.493
Myocardial infarction, *n* (%)	11 (2.0)	2 (1.1)	9 (2.5)	0.250
Stroke, *n* (%)	2 (0.3)	1 (0.5)	1 (0.2)	0.644
Deep vein thrombosis, *n* (%)	0 (0)	0 (0)	0 (0)	

The composite endpoint for thromboembolic complications includes pulmonary embolism, myocardial infarction, stroke, and deep vein thrombosis. Data are presented as absolute number (percentage) or mean ± SD. Bold face indicates *p*-values < 0.05.

## 4 Discussion

In this retrospective cohort study, we report that female sex was not correlated with the intraoperative administration of TXA during liver transplantation. The MELD-score was independently associated with the intraoperative administration of TXA. In our secondary endpoint analysis, graft failure, stroke, deep vein thrombosis, and pulmonary embolism within 30 days after liver transplantation occurred more frequently in women compared to men. In women, intraoperative administration of TXA was associated with a lower incidence of the composite endpoint for thromboembolic complications.

Gender-specific inequalities in the rate of liver transplantation exist. Fewer women than men are on the waiting list for liver transplants (16, 17). Alongside gender variations in the occurrence, progression, and consequences of chronic liver disease (18), a MELD score underestimated disease severity (19), an overestimation of renal function in women (19) and the limited availability of suitably sized livers due to women’s smaller physique (20, 21) seem to be influencing factors for women’s admission and ranking on the waiting list. Only around a third of all liver transplantations are performed on female patients (18). In this study, the proportion of women was 30%.

Gender specific differences in medical treatment can be observed in various medical areas (1, 2, 4, 22). In patients with out-of-hospital chest pain, women are less likely than men to receive recommended treatment, less likely to be transported to the hospital with sirens and blue lights, and less likely to be resuscitated in the event of circulatory arrest (2). In the treatment of pain, women receive opioids less frequently than men for conditions such as abdominal pain, headaches, flank pain, and trauma (1). Furthermore, women with bleeding trauma receive TXA treatment less frequently than men, despite the comparable reduction in mortality with TXA administration in both genders (4). The discrepancies in perioperative management during liver transplantation, especially concerning antifibrinolytic therapy, between women and male liver transplant recipients have not yet been investigated. No sex disparity in TXA treatment was found in this study. The absolute dose of TXA administered did not differ between the sexes. Interestingly, dosage of TXA per kilogram of body weight varied between sexes. Despite the average dose of TXA administered being consistent with the recommendations of the European guidelines for the treatment of severe bleeding (7), women received approximately 4.5 mg kg^−1^ more TXA than men. This can be attributed to a meanwhile outdated SOP, suggesting fixed amounts of TXA administration in adult patients rather than doses calculated based on body weight (9). Using TXA is still regarded critically today, as a high dose of TXA May elevate the risk of thromboembolic complications (6). In patients undergoing liver transplantation, a small number of case reports suggest a correlation between antifibrinolytic treatment and thromboembolic complications (23–25). Within our cohort of men as well as in several other studies and a meta-analysis, no correlation between TXA administration and thromboembolic complications was observed (5, 6, 9, 26–28). Interestingly, within the cohort of women, intraoperative administration of TXA was linked to a lower incidence of the composite endpoint for thromboembolic events. This observation is consistent with findings in previous publications (29). In bleeding trauma patients, TXA administration resulted in a lower rate of death from vascular occlusion and a significant reduction in non-fatal and fatal myocardial infarctions (29). Possible mechanisms underlying this antithrombotic effect of TXA are effects on plasmin-induced activation of platelets (30) and coagulation factors V and VIII (31) as well as inhibition of the inflammatory effect of plasmin (32, 33).

In total, the rate of thromboembolic events (deep vein thrombosis, stroke, and pulmonary embolism) was higher in women compared to men. Research on differences in postoperative complications after liver transplantation between women and men is limited. In line with our findings, in a review by Sarkar et al., overall survival after liver transplantation does not appear to differ according to sex (16). However, the underlying disease that led to the transplant appears to influence the occurrence of graft failure and mortality (16). For instance, in patients with hepatitis C, women face a heightened risk of graft loss (34–36). In patients with alcoholic liver disease or non-alcoholic steatohepatitis, gender did not impact the occurrence of graft failure (37–41). In our cohort, graft failure was more common in women than in men. In the subgroup analysis, where patients with hepatitis, alcoholic liver cirrhosis, malignant liver diseases, and cholangitis were examined separately, sex did not show a significant impact on graft failure rates or mortality. However, the sample sizes within these groups were small.

When evaluating the complication rate in our patient cohort, it is important to consider that, although women and men had similar MELD scores, women had a higher Child-Pugh score, were more frequently subjected to previous abdominal surgery, and were more often classified with high urgency on the waiting list on average, which might impact the outcome.

Our study has some limitation that warrant consideration. The retrospective study design limits the representativeness, validity, and reliability of the results. Furthermore, it lacks the capacity to fully control for unmeasured biases. The probability is low that all factors affecting the administration of tranexamic acid were thoroughly considered in the logistic regression analysis. The sample size was limited by the presence of digitalized anesthesia records. Moreover, the observation period was restricted. Prior to the new recommendation of the clinical practice guidelines (7), the utilization of tranexamic acid during liver transplantation had declined significantly since 2018 at our center. This decision was based on concerns about the risk of potential thrombotic events and on a study showing that hyperfibrinolysis was almost abolished 1 hour after graft reperfusion (42), suggesting that administering TXA during reperfusion might be negligible. In addition, although the administration of TXA is based on the evaluation of results of rotational thrombelastometry, these results were unavailable for a considerable number of patients because they were not digitized during the observation period and could therefore not be evaluated.

In conclusion, our main finding is that, different from other pre-hospital emergency scenarios (4), we did not find any sex-related differences in the administration of tranexamic acid during liver transplantations, an urgent but non-emergency setting under controlled in-hospital conditions. However, the results also suggest that women are at an increased risk of complications during liver transplantation. Likewise, it could be of clinical relevance that TXA administration might prevent woman from thromboembolic complications. Reasons underlying the observed sex bias rate remain uncertain and further research is warranted.

## Data Availability

The raw data supporting the conclusions of this article will be made available by the authors, without undue reservation.
