# Supplementary material for: Patient sex and use of tranexamic acid in liver transplantation
Source: Front Med (Lausanne). 2024 Sep 23;11:1452733. doi: 10.3389/fmed.2024.1452733 (PMC11456493; doi:10.3389/fmed.2024.1452733)
Supplement: Supplementary file 1 [file Table_1.DOCX]

**Supplemental Table 1: Applied algorithm for tranexamic acid administration in managing perioperative bleeding during liver transplantation, based on rotational thromboelastometry results in adult patients over the years**

|  | **Indication and dosage of tranexamic acid administration based on rotational thrombelastometry results** | | |
| --- | --- | --- | --- |
| **Years** | **2004-2007** | **2008-2011** | **2012-2017** |
|  | -CT_APTEM_ < CT_EXTEM_  Dosage: 1g | -CT_APTEM_ < CT_EXTEM_  -A10_APTEM_ > A10_EXTEM_  Dosage:20- 25mg kg^-1^ | -CT_APTEM_ < CT_EXTEM_  -A10_APTEM_ > A10_EXTEM_  - CLI30_EXTEM <_ 85%  Dosage: 20- 25mg kg^-1^ |

*A 10, amplitude at 10 min; CT, clotting time; CLI, clot lysis index.*
