# Supplementary material for: Patient sex and use of tranexamic acid in liver transplantation
Source: Front Med (Lausanne). 2024 Sep 23;11:1452733. doi: 10.3389/fmed.2024.1452733 (PMC11456493; doi:10.3389/fmed.2024.1452733)
Supplement: Supplementary file 2 [file Table_2.DOCX]

**Supplemental Table 2a:** **Underlying liver disease that led to liver transplantation**

| **Variable** | **Analysis set**  779 (100) | **Female sex**  234 (30) | **Male sex**  545 (70.0) | **p value** |
| --- | --- | --- | --- | --- |
| Alcoholic liver cirrhosis, n (%) | 211 (27.1) | 44 (18.8) | 167 (30.6) | **<0.01** |
| Metabolic liver diseases, n (%) | 33 (4.2) | 10 (4.3) | 23 (4.2) |  |
| Hepatitis, n (%) | 168 (21.6) | 38 (16.2) | 130 (23.9) |  |
| Acute liver failure, n (%) | 58 (7.4) | 33 (14.1) | 25 (4.6) |  |
| Malignant liver tumors, n (%) | 80 (10.3) | 17 (7.3) | 63 (11.6) |  |
| Biliary tract diseases, n (%) | 129 (16.6) | 46 (19.7) | 83 (15.2) |  |
| Cystic liver diseases, n (%) | 10 (1.3) | 8 (3.4) | 2 (0.4) |  |

| Amyloidosis, n (%) | 14 (1.8) | 8 (3.4) | 6 (1.1) |  |
| --- | --- | --- | --- | --- |
| Unspecified causes of liver cirrhosis  and other liver diseases (%) | 76 (9.8) | 30 (12.8) | 46 (8.4) |  |

**Supplemental Table 2b: Subgroup analysis: Postoperative complications**

| **Variable** | **Analysis set (n=779)** | **Female sex (n=234)** | **Male sex**  **(n=545)** | **p value** |
| --- | --- | --- | --- | --- |
| ***Alcoholic liver cirrhosis*** | 211 | 44 | 167 | **<0.001** |
| 30 -day- mortality, n (%) | 18 (8.5) | 5 (11.4) | 13 (7.8) | 0.450 |
| Graft failure, n (%) | 26 (12.3) | 9 (20.5) | 17 (10.2) | 0.065 |
| Pulmonary embolism, n (%) | 5 (2.4) | 2 (4.5) | 3 (1.8) | 0.286 |
| Myocardial infarction, n (%) | 6 (2.8) | 1 (2.3) | 5 (3.0) | 0.798 |
| Stroke, n (%) | 2 (0.9) | 1 (2.3) | 1 (0.6) | 0.308 |
| Deep vein thrombosis, n (%) | 0 (0) | 0 (0) | 0 (0) |  |
| Hepatic artery thrombosis, n (%) | 8 (3.8) | 4 (9.1) | 4 (2.4) | **0.039** |
| Portal vein thrombosis, n (%) | 6 (2.8) | 2 (4.5) | 4 (2.8) | 0.445 |
| Length of hospital stay (d), median (Q1, Q3) | 32 (22, 59) | 45 (19, 61) | 44 (23, 55) | 0.925 |
| Length of ICU stay (d), median (Q1, Q3) | 4 (2, 12) | 12 (2, 13) | 12 (2, 11) | 0.870 |
| ***Malignant liver tumors*** | 80 (10.3) | 17 (7.3) | 63 (11.6) | 0.070 |
| 30 -day- mortality, n (%) | 2 (2.5) | 0 (0) | 2 (3.2) | 0.457 |
| Graft failure, n (%) | 5 (6.3) | 2 (11.8) | 3 (4.8) | 0.290 |
| Pulmonary embolism, n (%) | 1 (1.3) | 0 (0) | 1 (1.6) | 0.601 |
| Myocardial infarction, n (%) | 1 (1.3) | 0 (0) | 1 (1.6) | 0.601 |
| Stroke, n (%) | 0 (0) | 0 (0) | 0 (0) |  |
| Deep vein thrombosis, n (%) | 0 (0) | 0 (0) | 0 (0) |  |
| Hepatic artery thrombosis, n (%) | 9 (11.3) | 3 (17.7) | 6 (9.5) | 0.519 |
| Portal vein thrombosis, n (%) | 1 (1.3) | 0 (0) | 1 (1.6) | 0.601 |
| Length of hospital stay (d), median (Q1, Q3) | 27 (21, 42) | 39 (22, 47) | 34 (20, 41) | 0.390 |
| Length of ICU stay (d), median (Q1, Q3) | 2 (1, 4) | 8 (1, 4) | 4 (1, 4) | 0.919 |
| ***Hepatitis*** | 168 (21.6) | 38 (16.2) | 130 (23.9) | **0.018** |
| 30 -day- mortality, n (%) | 6 (3.6) | 1 (2.6) | 5 (3.8) | 0.723 |
| Graft failure, n (%) | 16 (9.5) | 3 (7.9) | 13 (10) | 0.697 |
| Pulmonary embolism, n (%) | 2 (1.2) | 2 (5.3) | 0 (0) | **0.009** |
| Myocardial infarction, n (%) | 2 (1.2) | 0 (0) | 2 (1.5) | 0.442 |
| Stroke, n (%) | 1 (0.6) | 1 (2.6) | 0 (0) | 0.064 |
| Deep vein thrombosis, n (%) | 0 (0) | 0 (0) | 0 (0) |  |
| Hepatic artery thrombosis, n (%) | 10 (6.0) | 2 (5.3) | 8 (6.2) | 0.838 |
| Portal vein thrombosis, n (%) | 5 (3) | 3 (7.9) | 2 (1.5) | 0.072 |
| Length of hospital stay (d), median (Q1, Q3) | 28 (20, 47) | 40 (20, 47) | 40 (20, 45) | 0.982 |
| Length of ICU stay (d), median (Q1, Q3) | 3 (1, 8) | 8 (1, 6) | 7 (1, 8) | 0.847 |
| ***Biliary tract diseases*** | 129 (16.6) | 46 (19.7) | 83 (15.2) | 0.127 |
| 30 -day- mortality, n (%) | 8 (6.2) | 5 (10.9) | 3 (3.6) | 0.102 |
| Graft failure, n (%) | 9 (7) | 4 (8.7) | 5 (6) | 0.568 |
| Pulmonary embolism, n (%) | 0 (0) | 0 (0) | 0 (0) |  |
| Myocardial infarction, n (%) | 1 (0.8) | 1 (2.2) | 0 (0) | 0.177 |
| Stroke, n (%) | 0 (0) | 0 (0) | 0 (0) |  |
| Deep vein thrombosis, n (%) | 2 (1.6) | 2 (4.3) | 0 (0) | 0.056 |
| Hepatic artery thrombosis, n (%) | 5 (3.9) | 3 (6.5) | 2 (2.4) | 0.475 |
| Portal vein thrombosis, n (%) | 6 (4.7) | 2 (4.3) | 4 (4.8) | 0.742 |
| Length of hospital stay (d), median (Q1, Q3) | 30 (20, 50) | 42 (22, 50) | 37 (19, 47) | 0.106 |
| Length of ICU stay (d), median (Q1, Q3) | 2 (1, 6) | 8 (1, 8) | 5 (1, 6) | 0.188 |

Data are presented as absolute numbers (percentage) or mean ± SD. Bold face indicates p-values < .05.

*ICU, intensive care unit; SD, standard deviation.*
