# Supplementary material for: Patient sex and use of tranexamic acid in liver transplantation
Source: Front Med (Lausanne). 2024 Sep 23;11:1452733. doi: 10.3389/fmed.2024.1452733 (PMC11456493; doi:10.3389/fmed.2024.1452733)
Supplement: Supplementary file 3 [file Table_3.DOCX]

**Supplemental Table 3: Intraoperative transfusions and coagulation factor administrations**

| **Variable** | **Analysis set** | **Women** | **Men** | **p-value** |
| --- | --- | --- | --- | --- |
|  | 779 (100) | 234 (30) | 545 (70.0) |  |
| RBC, n (%),  (TU) Mean ± SD | *n=581 (74.6)*  8.4 ± 8.4 | *n= 175 (74.8)*  7.8±7.3 | *n=406 (74.5)*  8.7±8.8 | 0.301 |
| FFP (TU), n (%),  Mean ± SD | *n=698 (89.6)*  17.3 ± 12.2 | *n=203 (86.8)*  15.7±11.3 | *n=495 (90.8)*  18.0±12.5 | **0.014** |
| Platelets, n (%),  (TU) Mean ± SD | *n=523 (67.1)*  3.2 ± 2.2 | *n=153 (65.4)*  3.3±2.6 | *n=370 (67.9)*  3.2±2.0 | 0.910 |
| PCC, n (%),  (IU), mean ± SD | *n=291 (37.4)*  3.561 ± 2.482 | *n=96 (41.0)*  3.7±2.9 | *n=195 (35.8)*  3.5±2.3 | 0.668 |
| Fibrinogen concentrate, n (%),  (g), Mean ± SD | *n= 404 (51.9)*  4.8 ± 3.3 | *n=123 (52.6)*  4.50±3.3 | *n=281 (51.6)*  5.0±3.4 | 0.141 |

Data are presented as mean ± SD.

*SD, standard deviation; IU, international units; RBC, red blood cell concentrate; FFP, fresh frozen plasma; PCC, prothrombin complex concentrate.*
