# Supplementary material for: Patient sex and use of tranexamic acid in liver transplantation
Source: Front Med (Lausanne). 2024 Sep 23;11:1452733. doi: 10.3389/fmed.2024.1452733 (PMC11456493; doi:10.3389/fmed.2024.1452733)
Supplement: Supplementary file 4 [file Table_4.DOCX]

**Supplemental Table 4: Clinical baseline characteristics of the study cohort** **compared in relation to intraoperative administration of tranexamic acid**

| **Variable** | **Analysis set**  779 (100) | **Intraoperative TXA**  262 (33.6) | **No intraoperative TXA**  517 (66.4) | **p value** |
| --- | --- | --- | --- | --- |
| ***Recipient*** |  |  |  |  |
| Female Sex, n (%) | 234 (30) | 74 (28.2) | 160 (30.9) | .437 |
| Age (y), mean ± SD | 52.2 ± 10.3 | 52.75 ± 9.72 | 51.912 ± 10.63 | .504 |
| Weight (kg), mean ± SD | 80.2 ± 16.6 | 81.225 ± 16.43 | 79.601 ± 16.62 | .213 |
| BMI (kg m^-2^), mean ± SD | 26.6 ± 4.8 | 26.637 ± 4.64 | 26.503 ± 4.89 | .699 |
| Retransplantation, n (%) | 67 (8.6) | 35 (13.3) | 32 (6.1) | **<.001** |
| *Child-Pugh, n (%)* |  |  |  |  |
| A | 221 (28.4) | 62 (23.7) | 159 (30.8) | .116 |
| B | 214 (27.5) | 77 (29.4) | 137 (26.5) | . |
| C | 344 (44.2) | 123 (46.9) | 221 (42.7) |  |
| MELD, mean ± SD | 19.3 ± 10.4 | 21.33 ± 11.07 | 18.21 ± 9.84 | **<.001** |
| ***Donor*** |  |  |  |  |
| Age (y), mean ± SD | 58.1 ± 17.3 | 58.65 ± 17.539 | 57.82 ± 17.252 | .570 |
| Female sex, n (%) | 359 (46.1) | 112 (42.7) | 247 (47.8) | .184 |
| Weight (kg), mean ± SD | 77.5 ± 15.0 | 78.61 ± 14.66 | 76.91 ± 15.21 | .208 |
| BMI (kg m^-2^), mean ± SD | 26.3 ± 6.1 | 27.01 ± 8.575 | 25.974 ± 4.207 | .117 |
| Weight ratio recipient/donor, mean ± SD | 1.05 ± 0.24 | 1.051 ± .228 | 1.056 ± .246 | .704 |
| ***Urgency, n (%)*** |  |  |  |  |
| T | 736 (94.5) | 251 (95.8) | 485 (93.8) | .250 |
| HU | 43 (5.5) | 11 (4.2) | 32 (6.2) |  |
| ***Medical history, n (%)*** |  |  |  |  |
| Previous deep vein thrombosis | 11 (1.4) | 1 (0.3) | 10 (1.9) | .083 |
| Previous hepatic artery thrombosis | 11 (1.4) | 5 (1.9) | 6 (1.16) | .403 |
| Previous portal vein thrombosis | 84 (10.8) | 32 (12.2) | 52 (10.1) | .359 |
| Other thrombosis in history | 107 (13.7) | 45 (17.2) | 62 (11.2) | **.047** |
| Previous abdominal surgery, n (%) | 275 (35.3) | 97 (37.0) | 178 (34.4) | .474 |

| Coronary heart disease, n (%) | 66 (8.47) | 27 (10.3) | 39 (7.5) | .191 |
| --- | --- | --- | --- | --- |
| History of myocardial infarction, n (%) | 20 (2.567) | 8 (3.1) | 12 (2.3) | .541 |
| History of stroke, n (%) | 6 (0.77) | 3 (1.1) | 3 (0.6) | .394 |

| ***Surgery data*** |  |  |  |  |
| --- | --- | --- | --- | --- |
| Preoperative hemoglobin (g/dl), mean ± SD | 10.9 ± 2.6 | 10.5 ± 2.7 | 11.1 ± 2.5 | **<.001** |
| Preoperative platelet count, mean ± SD | 119.5 ± 91.3 | 105.4 ± 80.7 | 126.6 ± 95.5 | **<.001** |
| Bood loss (ml/kg), mean ± SD | 55.3 ±57.6 | 64.1 ±64.6 | 50.9±53.3 | **.002** |
| Cold ischemia time (h), mean ± SD | 8.6 ± 3.1 | 8.83 ± 3.17 | 8.47 ± 3.01 | **.010** |
| Surgery time (h), mean ± SD | 5.8 ± 1.5 | 6.01 ± 1.345 | 5.69 ± 1.508 | **.004** |

Data are presented as mean ± SD, or as absolute number (percentage).

*SD, standard deviation; BMI, body mass index; MELD, Model for End-stage Liver Disease; T, transplantable; HU, high urgency.*
